# Serum Irisin Levels, Endothelial Dysfunction, and Inflammation in Pediatric Patients with Type 2 Diabetes Mellitus and Metabolic Syndrome

**DOI:** 10.1155/2020/1949415

**Published:** 2020-09-04

**Authors:** Anna S. Huerta-Delgado, Daniel N. Roffe-Vazquez, Adrian M. Gonzalez-Gil, José R. Villarreal-Calderón, Oscar Tamez-Rivera, Nora A. Rodriguez-Gutierrez, Elena C. Castillo, Christian Silva-Platas, Gerardo Garcia-Rivas, Leticia Elizondo-Montemayor

**Affiliations:** ^1^Center for Research in Obesity and Clinical Nutrition, Tecnologico de Monterrey-Escuela de Medicina, Monterrey 64710, Mexico; ^2^Department of Pediatrics, Tecnologico de Monterrey-Hospital Zambrano Hellion, San Pedro Garza-Garcia 66278, Mexico; ^3^Department of Pediatrics, Hospital Regional de Alta Especialidad Materno-Infantil, Guadalupe 67140, Mexico; ^4^Center for Biomedical Research, Tecnologico de Monterrey-Hospital Zambrano Hellion, San Pedro Garza-Garcia 66278, Mexico; ^5^Cardiovascular Medicine and Metabolomics Research Group, Tecnologico de Monterrey-Hospital Zambrano Hellion, San Pedro Garza-Garcia 66278, Mexico

## Abstract

The prevalence of type 2 diabetes mellitus (T2DM) and metabolic syndrome (MetS) has increased in the pediatric population. Irisin, an adipomyokine, is involved in white adipose tissue browning, energy expenditure, insulin sensitivity, and anti-inflammatory pathways. Data on the associations among circulating irisin levels, soluble cell adhesion molecules (sCAMs), and inflammatory cytokines is scarce in children and adolescents with MetS and T2DM. Subjects aged 6-16 years were grouped into T2DM, MetS, and healthy controls. Serum irisin levels were significantly lower in the MetS (6.6 [2.8-18.0] ng/mL) and T2DM (6.8 [2.2-23.2] ng/mL) groups compared with controls (30.3 [24.6-57.1] ng/mL). Negative correlations between irisin and the BMI percentile (*R* = −0.358), WC percentile (*R* = −0.308), and triglycerides (*R* = −0.284) were identified, while positive associations with TC (*R* = 0.287), HDL-c (*R* = 0.488), and LDL-c (*R* = 0.414) were observed. Significant negative correlations were found between irisin and sNCAM (*R* = −0.382), sICAM-2 (*R* = −0.300), sVCAM-1 (*R* = −0.292), MCP-1 (*R* = −0.308), and IFN-*α*2 (*R* = −0.406). Of note, lower concentrations of most sCAMs (sICAM-1, sPSGL-1, sP-selectin, sEpCAM, sICAM-2, sALCAM, sPECAM-1, sCD44, sVCAM-1, sICAM-3, sL-selectin, and sNCAM) were shown in T2DM subjects compared with MetS patients. Lower irisin levels induce a lack of inhibition of oxidative stress and inflammation. In T2DM, higher ROS, AGEs, glucotoxicity, and inflammation trigger endothelial cell apoptosis, which downregulates the sCAM expression as a compensatory mechanism to prevent further vascular damage. In opposition, in subjects with MetS that have not yet developed T2DM and its accompanying stressors, the upregulation of the sCAM expression is ensued.

## 1. Introduction

Childhood obesity and its related disorders, including type 2 diabetes mellitus (T2DM) and metabolic syndrome (MetS), are worldwide growing public health problems. According to the World Health Organization (WHO), in 2016, more than 340 million children and adolescents aged 5 to 19 years old were either overweight or obese (defined as body mass index- (BMI-) for age >1 or >2 standard deviations, respectively, above the WHO growth reference median) [1]. Additionally, the prevalence and incidence of MetS [2] and T2DM [3] have also increased in the pediatric population. For instance, the reported prevalence of MetS among adolescents in the United States increased from 4.2% in the National Health and Nutrition Examination Survey (NHANES) III to 6.4% in NHANES IV [4], while the incidence of T2DM amongst subjects 10 to 19 years old increased from 9.0 per 100,000 individuals per year in 2003 to 13.8 per 100,000 individuals per year in 2015 [5]. Moreover, cardiovascular disease, responsible for premature morbidity and mortality, is initiated and maintained by the chronic metabolic and inflammatory alterations present in these diseases [6].

Indeed, the low-grade systemic inflammation in children with MetS and T2DM, characterized by the augmented levels of tumor necrosis factor-alpha (TNF-*α*), interferon-gamma (IFN-*γ*), interleukin- (IL-) 1, and IL-6, has been previously described [7]. The hypertrophic adipose tissue has been shown to promote the downstream activation of nuclear factor-kappa B (NF-*κ*B) via multiple pathways including toll-like receptor 4 (TLR4) activation, mechanical stress, and hypoxia [8]. Subsequently, increased expression of inflammation drivers ensues, including inflammatory cytokines, chemokines, intercellular adhesion molecules (ICAMs), and vascular cell adhesion molecules (VCAMs), among others [9]. In addition, endothelial dysfunction is considered an initiating event of vascular diseases [10], starting early in childhood in patients with obesity, MetS, and T2DM, and has been strongly associated with a proinflammatory state [11]. For instance, higher levels of soluble E-selectin (sE-selectin), sVCAM-1, and sICAM-1 were found in obese children and adolescents [11, 12], in those with insulin resistance and MetS [13, 14], and in adolescents and young adults with T2DM [15].

Understanding the pathways involved in the inflammatory and vascular complications that accompany obesity, MetS, and T2DM in the pediatric population has become an important research topic. In this regard, irisin, a novel adipomyokine secreted mainly by the skeletal muscle following acute bouts of exercise [16, 17], and to a lesser extent by adipose tissue, might contribute to these pathogenic changes [18, 19]. It was first identified in exercised mice overexpressing the *Ppargc1a* gene, resulting in increased transcription of the peroxisome proliferator-activated receptor-*γ* coactivator 1-*α* (PGC-1*α*) protein [16]. PGC-1*α* has been described to promote the expression of the transmembrane fibronectin type III domain-containing protein 5 (FNDC5), which releases the 112-amino acid peptide irisin [20]. This adipomyokine promotes uncoupling protein-1 (UCP1) expression in adipocytes and thus “browning” of white adipose tissue, increased energy expenditure, thermogenesis, and improved insulin sensitivity [16]. Preclinical studies have described direct protective effects on the vascular endothelium in the context of obesity [21], T2DM [22], and hypertension [23]. Importantly, irisin has also shown anti-inflammatory properties in macrophages and adipocytes [24–28], and it has been described to favor glucose uptake [29].

Most of the clinical studies in adults have demonstrated increased irisin levels and positive associations with adiposity parameters in patients with obesity [30]. In obese patients diagnosed with MetS, both higher [31] and lower [32] irisin concentrations have been described when compared with those without MetS. On the other hand, in adult populations with T2DM, lower irisin levels have been reported when compared with healthy controls [33]. In the pediatric population, specifically regarding children with MetS and T2DM, reports are either controversial or scarce [34]. For instance, positive correlations between plasma irisin concentration and both triglyceride levels and the Homeostatic Model Assessment (HOMA) index were described in nondiabetic children [35] and adolescents with MetS [36]. On the contrary, lower serum irisin concentrations were shown in overweight and obese children with MetS compared with those without MetS [37, 38]. Some authors have suggested that changes in the concentration of irisin might play a role in the pathogenesis of MetS [31–33, 36–38]. Noteworthy, a study in pediatric patients with T2DM reported decreased levels of irisin when compared with healthy controls [39]. In addition, evidence concerning the hypothesized role of irisin in endothelial function and inflammation is scarce in the pediatric population. Increased plasma levels of adhesion molecules, alongside lower plasma irisin concentrations and negative correlations between irisin and E-selectin, ICAM-1, and TNF-*α* were found in obese children when compared with lean controls [40]. Furthermore, an increase in endothelial progenitor cells and plasma irisin levels was shown in overweight/obese children, which was attributed to a compensatory mechanism elicited in an attempt to repair vascular damage [35].

Overall, the role of irisin remains understudied and controversial in the pediatric population, especially in the context of MetS and T2DM. Data on the relationship of this adipomyokine with the endothelial dysfunction and inflammation is limited in this age group, highlighting the importance of providing an insight into the understanding of its potential role in the pathophysiology of these diseases. In addition, there are no studies, to our knowledge, describing the association between circulating irisin levels, a broad panel of soluble cell adhesion molecules (CAMs), and a comprehensive panel of inflammatory cytokines in the pediatric population of both patients with MetS and T2DM. Therefore, the aim of this study is to determine the relationship of irisin with a broad panel of soluble markers of endothelial dysfunction and inflammatory cytokines, as well as biochemical parameters in children and adolescents with T2DM, MetS, and healthy controls.

## 2. Materials and Methods

### 2.1. Study Population

We performed a cross-sectional study in a cohort of 56 pediatric patients aged 6-16 years old. The subjects were grouped into T2DM (11 girls and 10 boys), MetS without T2DM (8 girls and 11 boys), and healthy controls (9 girls and 8 boys). All patients were of Mexican origin living in the northeastern region of this country. Patients classified as T2DM met at least one of the four criteria defined by the American Diabetes Association [41]: (1) glycated hemoglobin (Hb1Ac) ≥ 6.5%, (2) fasting plasma glucose ≥ 126 mg/dL, (3) 2 − hour plasma glucose ≥ 200 mg/dL during an oral glucose tolerance test with 1.75 g/kg of glucose (75 g maximum), or (4) random plasma glucose ≥ 200 mg/dL in a patient with symptoms of hyperglycemia or a hyperglycemic crisis [41]. Patients in the MetS group met at least three criteria according to Cook et al.'s modified definition of MetS: (1) triglycerides ≥ 110 mg/dL [42], (2) high − density lipoprotein cholesterol (HDL − c) levels ≤ 40 mg/dL (for both boys and girls) [42], (3) waist circumference (WC) ≥ 90^th^ percentile for age and sex [43], (4) fasting glucose levels ≥ 100 mg/dL and ≤ 125 mg/dL [44] and/or the HOMA index ≥ 2.5 [45], or (5) systolic or diastolic arterial blood pressure ≥ 90^th^ percentile for age, sex, and height and/or treatment with any antihypertensive medication [46]. Patients in the MetS group were excluded if they met the diagnostic criteria for T2DM. The healthy control group consisted of children with normal weight-for-height, weight-for-age, and BMI-for-age values, according to the Centers for Disease Control and Prevention (CDC) criteria for boys and girls [47]. They were excluded from this group if they presented any previous medical conditions and metabolic abnormalities (including dyslipidemias and/or glucose alterations) or used antihypertensive medication. The protocol was thoroughly explained to patients and their legal guardians, written informed consent was obtained from the latter, and verbal assent was given by the children. Patients diagnosed with T2DM and MetS were recruited from the Hospital Regional de Alta Especialidad Materno Infantil within a period of 2 years. Healthy controls were also recruited during a 2-year period from the outpatient routine pediatric clinics. The Ethics and Research Commissions of the School of Medicine of Tecnologico de Monterrey approved this protocol under the code “AIEMPPDM2SM,” while the Mexican Secretariat of Health gave its approval with the Bioethics National Commission code “CONBIOETICA-19-CEI-011-20161017” and the Ethics Committee code “13CI19039138.”

### 2.2. Anthropometric and Clinical Parameters

Standardized protocols were followed to obtain weight (in kilograms), height (in centimeters), WC (in centimeters), BMI (in kg/m^2^), and fat mass (in grams) [43]. Height was measured with a stadiometer (SECA® 217, SECA Mexico, Mexico City, Mexico). A bioimpedance technique with a TANITA® BF-689 scale (Tanita Corporation of America, Inc., Arlington Heights, IL, USA) was used to measure body fat percentage (BF%). These values were interpreted according to the CDC weight-for-age, weight-for-height, height-for-age, and WC-for-age percentile charts for boys and girls [47]. The BMI percentile for sex and age was calculated according to the CDC's BMI Percentile Calculator for Child and Teen [48]. Tanner's scale was used to determine every patient's pubertal stage. Systolic and diastolic blood pressures (SBP and DBP, respectively) were registered with the patient in a seated position and at rest. Blood pressure measurements were obtained in triplicate using a mercury sphygmomanometer with an appropriate cuff size. SBP and DBP percentiles were obtained according to the Fourth Report on the Diagnosis, Evaluation, and Treatment of High Blood Pressure in Children and Adolescents [49].

### 2.3. Biochemical Parameters

Peripheral venipuncture after an overnight 12-hour fasting was performed to withdraw blood samples of patients. The samples were immediately processed to collect serum and plasma by centrifugation and were then stored at -80°C. Fasting glucose levels were obtained using the hexokinase/glucose-6-phosphate dehydrogenase method with the Glucose 3L82 (304772/R02) reagent kit, with intra- and interassay coefficients of variation (CV) of ≤5% (Denka Seiken Co. Ltd., Tokyo, Japan), while serum insulin concentrations were measured via chemiluminescence using the Architect Insulin 8K41-27 reagent kit, with intra- and interassay CV of ≤7% (Abbott Laboratories Diagnostic Division, IL, USA), both determined with the Architect cSystem. Glycated hemoglobin (HbA1c) was obtained with a high-resolution liquid chromatography on the Variant II (BioRad D10®) system, with intra- and interassay CV of ≤5%. The HOMA index was calculated using the Matthews formula [fasting insulin (mIU/mL) × fasting glucose (mg/dL)/405] [50]. Triglycerides were measured by the glycerol-phosphate-oxidase reaction using the Triglyceride 7D74-20 (30-3140/R3) reagent kit, with intra- and interassay CV of ≤5% (Abbott Laboratories Diagnostic Division, Chicago, IL, USA) using the Architect cSystem. The Cholesterol 7D62 (304796/R02) reagent kit, with intra- and interassay CV of ≤3% (Abbott Laboratories Diagnostic Division, Chicago, IL, USA), was used to obtain total cholesterol (TC) concentrations with an enzymatic assay using the Architect cSystem. Through an accelerator selective detergent method, HDL-c was determined by the Ultra HDL 3K33-21 (306571/R03) assay kit, with intra- and interassay CV of ≤4% (Abbott Laboratories Diagnostic Division, Chicago, IL, USA) utilizing the Architect cSystem. Low-density lipoprotein cholesterol (LDL-c) was calculated from the triglyceride, TC, and HDL-c values using Friedewald's formula [(TC)–(HDLc) − (triglycerides/5)] [51].

### 2.4. Serum Irisin

The serum irisin concentration was determined by the sandwich enzyme-linked immunosorbent assay (ELISA) using a human irisin ELISA kit (SK00170-08) (Aviscera Bioscience Inc., Santa Clara, CA, USA). This assay's sensitivity is 75-100 pg/mL, with intra- and interassay coefficients of variation of 4-6% and 8-10%, respectively. It has a standard curve linear range of 0.8-51.2 ng/mL.

### 2.5. Adhesion Molecule and Cytokine Levels

A Human Adhesion Molecule Panel (13-plex) was used in order to determine the concentration of soluble cell adhesion molecules in plasma using the LEGENDplex^TM^ Multi-Analyte Flow Cytometry Assay Kit (cat. No. 740945) (BioLegend®, San Diego, CA, USA) for the indirect assessment of the endothelial dysfunction. The soluble molecules measured, along with their intra- and interassay coefficients of variation (respectively), included the following: sICAM-1 (CV 4-7%, 8-15%), soluble P-selectin glycoprotein ligand-1 (sPSGL-1) (CV 5-7%, 9-18%), sE-selectin (CV 4-6%, 5-16%), soluble P-selectin (sP-selectin) (CV 5-7%, 7-17%), soluble epithelial cell adhesion molecule (sEpCAM) (CV 5-7%, 7-15%), sICAM-2 (CV 5-8%, 6-17%), soluble activated leukocyte cell adhesion molecule (sALCAM) (CV 6-9%, 7-19%), soluble platelet endothelial cell adhesion molecule (sPECAM-1) (CV 3-7%, 7-16%), CD44 (CV 2-5%, 5-12%), sVCAM-1 (CV 5-7%, 10-18%), sICAM-3 (CV 6-7%, 11-15%), soluble L-selectin (sL-selectin) (CV 5-6%, 13-14%), and soluble neural cell adhesion molecule (sNCAM) (CV 3-7%, 8-12%). A Human Inflammation Panel (13-plex) was used to quantify plasma soluble cytokine levels with the LEGENDplex^TM^ Multi-Analyte Flow Cytometry Assay Kit (cat. No. 740118) (BioLegend®, San Diego, CA, USA). The cytokines measured, along with their intra- and interassay coefficients of variation (respectively), included IL-1*β* (CV 3.5-3.7%, 13.2-16.0%), IFN-*α*2 (CV 3.6-3.9%, 20.1-23.9%), IFN-*γ* (CV 3.0%-3.3%, 12.6-18.4%), TNF-*α* (CV 3.2-3.4%, 11.1-13.3%), monocyte chemoattractant protein-1 (MCP-1) (CV 2.5-2.6%, 8.3-8.4%), IL-6 (CV 2.8-3.5%, 20.2-20.5%), IL-8 (CV 2.9-3.0%, 11.0-16.9%), IL-10 (CV 2.6-2.7%, 9.1-12.7%), IL-12p70 (CV 3.2%, 11.5-14.1%), IL-17A (CV 2.1-2.4%, 7.6-21.4%), IL-18 (CV 3.3%, 6.6-7.6%), IL-23 (CV 2.6-3.3%, 9.3-9.8%), and IL-33 (CV 3.6-4.3%, 19.4-21.9%). The FACSCanto II (Becton Dickinson, Franklin Lakes, NJ, USA) flow cytometer was used for both the plasma soluble adhesion molecule and inflammatory cytokine panel measurements. According to BioLegend®'s recommendation, each measurement was performed in triplicate. The concentration for each analyte was obtained using the standard curves provided and the LEGENDplex^TM^ v8.0 software (license: [a6d7e4db-f091-4109-a70e-5ed3b61c2716]).

### 2.6. Statistical Analysis

The Prism GraphPad (version 8.0, GraphPad Software; San Diego, CA, USA) and the open-source R platforms (R package version 3.6.1; The R Foundation, Vienna, Austria), using packages “Hmisc” [52], “PMCMRplus” [53], and “ComplexHeatmap” [54], were used to carry out the statistical analyses [55]. To assess the normality of the sample's distribution, Shapiro-Wilk tests were performed for each variable. To compare the three groups when analysing adhesion molecules and cytokine profiles, Kruskal-Wallis with Dunn's multiple comparison tests was employed for nonparametric data, while ordinary one-way ANOVA with Holm-Sidak's multiple comparison tests was used for parametric data. For the rest of the variables, Kruskal-Wallis with Nemenyi's tests was applied for nonparametric data, while ordinary one-way ANOVA with Tukey's honest significant difference tests was used for the parametric data for comparison among groups. Chi-squared test and Fisher's exact test were applied to compare categorical variables among the three groups. The correlation coefficients for variables were assessed with Spearman's rank correlation test. Multivariate logistic regression analyses were performed to evaluate the age and sex variables as potential confounders. The Prism GraphPad v8.0 software was used with the following license: ACTGP-7ACDA3BF-DC092574-6A27F761-C26AE4D0. The BioRender® platform (BioRender, Toronto, ON, Canada) was used with a premium member's account (A01191497@itesm.mx) to create an original figure.

## 3. Results

### 3.1. Demographic and Anthropometric Characteristics

The demographic and anthropometric characteristics of the studied population are presented in Table 1. No significant differences of sex distribution among the groups were identified. The age difference between the T2DM group (13.8 ± 1.5 years) and healthy controls (11.4 ± 2.8 years) was noted (*p* = 0.004), while no age difference was observed between the MetS patients (12.4 ± 2.1 years) and the other two groups. Additionally, most of the children from the healthy control group were classified as Tanner stage 1 (37.5%), while children from the T2DM group were mainly classified as Tanner stage 4 (42.9%) (*p* = 0.006); no difference was seen between the MetS patients and the other two groups. The median BMI of both the MetS (26.0) and T2DM (27.7) groups were significantly higher compared with that of the healthy controls (17.8) (*p* < 0.001). This statistical difference was preserved when considering the median BMI percentile of the MetS (98^th^ percentile) and T2DM (96^th^ percentile) groups compared with healthy controls (60^th^ percentile) (*p* < 0.001), although no difference was seen between the MetS and T2DM patients. These results were independent of sex and age. A similar pattern was seen regarding WC, as the MetS (89.0 ± 12.4 cm) and T2DM (95.7 ± 16.4 cm) groups presented higher values when compared with healthy controls (64.1 ± 6.0 cm) (*p* < 0.001). The MetS (85^th^ percentile) and T2DM (85^th^ percentile) groups also showed higher median values of the WC percentile compared with healthy controls (25^th^ percentile) (*p* < 0.001). These findings were also independent of sex and age. A tendency towards a higher median SBP percentile in both the MetS and T2DM groups (79^th^ and 62^nd^ percentile, respectively) compared with the healthy controls group (38^th^ percentile) was found, although it did not reach statistical significance (*p* = 0.053).

### 3.2. Biochemical Parameters

**Figure 1:**
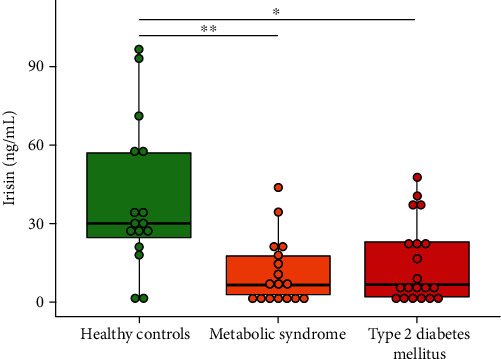
Serum irisin concentration in children and adolescents with type 2 diabetes mellitus, metabolic syndrome, and healthy controls. Healthy controls (30.3 ng/mL [24.6-57.1 ng/mL]), metabolic syndrome (6.6 ng/mL [2.8-18.0 ng/mL]), and type 2 diabetes mellitus (6.8 ng/mL [2.2-23.2 ng/mL]). Data are presented as median (interquartile range) for nonparametric data. ^∗^*p* < 0.05; ^∗∗^*p* < 0.01.

The biochemical parameters of the three study groups are shown in Table 2. The serum irisin concentration was lower in both the MetS and T2DM groups (6.6 ng/mL [2.8-18.0 ng/mL] and 6.8 ng/mL [2.2-23.2 ng/mL], respectively), compared with the controls (30.3 ng/mL [24.6-57.1 ng/mL]) (*p* = 0.004) (Figure 1). The observed differences of irisin between the three groups were independent of sex and age. When comparing with healthy controls (87.0 mg/dL [79.0-89.0 mg/dL]), glucose levels were higher in the MetS (94.0 mg/dL [89.5-103.5 mg/dL]) and T2DM (115.0 mg/dL [92.0-169.0 mg/dL]) groups (*p* < 0.001). Insulin shared this pattern, with higher concentrations in both the MetS (20.1 mIU/L [15.3-25.8 mIU/L]) and T2DM (22.9 mIU/L [10.6-29.8 mIU/L]) groups in comparison with the healthy controls (6.6 mIU/L [5.4-7.9 mIU/L]) (*p* < 0.001). On the other hand, HOMA index levels were significantly higher in patients with T2DM (9.1 ± 7.0) versus those with MetS (5.4 ± 2.2) and healthy controls (1.3 ± 0.4) (*p* < 0.001), while the MetS group also differed from the control group. These results were independent of sex and age for the three study groups. Higher levels of triglycerides were also found in patients with T2DM (154.0 mg/dL [112.0-187.0 mg/dL]) and MetS (144.0 mg/dL [128.0-213.5 mg/dL]) versus controls (90.0 mg/dL [65.8-98.5 mg/dL]) (*p* < 0.001). In addition, HDL-c was shown to be higher in healthy controls (53.8 ± 13.0 mg/dL) as opposed to the MetS (38.6 ± 7.8 mg/dL) and T2DM (37.2 ± 7.9 mg/dL) groups (*p* < 0.001). The differences regarding triglycerides and HDL-c were also independent of sex and age. In contrast, LDL-c levels were lower in the T2DM group (75.9 ± 20.6 mg/dL) when compared with the control group (98.6 ± 23.8 mg/dL) (*p* = 0.021).

### 3.3. Endothelial Dysfunction Markers

The results for levels of soluble cell adhesion molecules are summarized in Table 3. sICAM-1, sPSGL-1, sEpCAM, sICAM-2, sALCAM, sCD44, sVCAM-1, sICAM-3, sL-selectin, and sNCAM circulating levels were significantly lower in patients from both the healthy controls and T2DM groups when compared with the MetS group (from *p* = 0.015 to *p* < 0.0001). The higher levels of adhesion molecules in the MetS group were independent of sex and age. The T2DM group also presented significantly lower levels of sP-selectin and sPECAM-1 when compared with the MetS group (*p* = 0.018 and *p* = 0.002, respectively), but not with the healthy controls. sE-Selectin levels were significantly higher in the MetS patients when compared with healthy controls (*p* = 0.024), while no difference with the T2DM patients was observed. There were no significant differences between the healthy controls and the T2DM groups for any of the soluble adhesion molecules.

### 3.4. Cytokine Profile

The cytokine profile of the three study groups is shown in Table 4. The group with T2DM presented significantly higher levels of MCP-1 and IL-18 when compared with the control group (*p* = 0.011 and *p* = 0.003, respectively); these results were independent of sex and age. There was no statistical difference between groups for the rest of the cytokines, although there was a slight tendency towards higher plasma cytokine levels in both the MetS and T2DM groups when compared with healthy controls.

### 3.5. Correlation of Irisin with Anthropometric Characteristics, Biochemical Parameters, Cytokine Profile, and Adhesion Molecules

**Figure 2:**
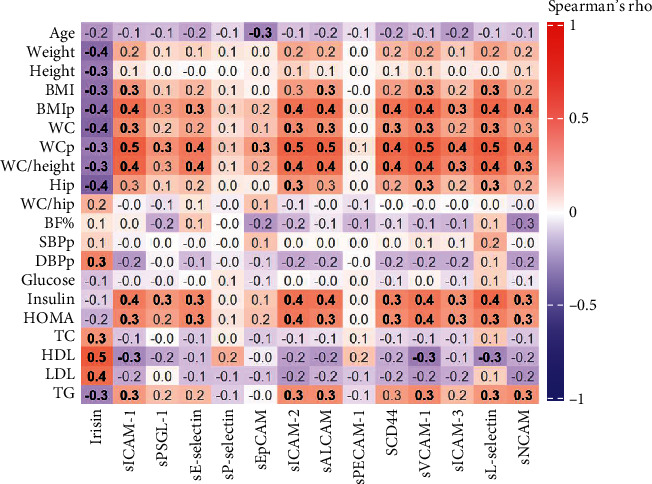
Correlation heat map for irisin and adhesion molecules with anthropometric and biochemical parameters in children and adolescents with type 2 diabetes mellitus, metabolic syndrome, and healthy controls. Colored squares of the heat map represent Spearman's rho correlation coefficients; the coefficients' font appears black and bold when correlations are statistically significant and grey when they are not. Squares filled in shades of blue represent negative correlations, while those filled in shades of red represent positive correlations. Statistical significance was established as *p* value ≤ 0.05. BMI: body mass index; BMIp: BMI percentile; WC: waist circumference; WCp: waist circumference percentile; BF%: body fat percentage; SBPp: systolic blood pressure percentile; DBPp: diastolic blood pressure percentile; HOMA: Homeostatic Model Assessment; TC: total cholesterol; HDL: high-density lipoprotein cholesterol; LDL: low-density lipoprotein cholesterol; TG: triglycerides; sICAM: soluble intercellular adhesion molecule; sPSGL: soluble P-selectin glycoprotein ligand; sEpCAM: soluble epithelial cell adhesion molecule; sALCAM: soluble activated leukocyte cell adhesion molecule; sPECAM: soluble platelet endothelial cell adhesion molecule; sVCAM: soluble vascular cell adhesion molecule; sNCAM: soluble neural cell adhesion molecule.

When analysing all three groups as a whole (Figure 2), a negative correlation was found between irisin and the BMI percentile (*R* = −0.358, *p* = 0.008), WC percentile (*R* = −0.308, *p* = 0.025), weight (*R* = −0.360, *p* = 0.008), and WC-to-height ratio (*R* = −0.321, *p* = 0.019). On the other hand, a positive correlation was observed between irisin and TC (*R* = 0.287, *p* = 0.037), HDL-c (*R* = 0.488, *p* < 0.0001), and LDL-c (*R* = 0.414, *p* = 0.002), while a negative correlation was obtained with triglycerides (*R* = −0.284, *p* = 0.039). No significant correlation was found between irisin and glucose, insulin, or the HOMA index. The irisin concentration was independent of the Tanner stage for the three groups. Additionally, adhesion molecules sICAM-1, sE-selectin, sICAM-2, sALCAM, sCD44, sVCAM-1, sICAM-3, sL-selectin, and sNCAM correlated positively with the BMI percentile, WC percentile, and WC-to-height ratio (*R* = 0.3 to *R* = 0.5, *p* < 0.05), as well as with insulin levels and the HOMA index (*R* = 0.3 to *R* = 0.4, *p* < 0.05).

**Figure 3:**
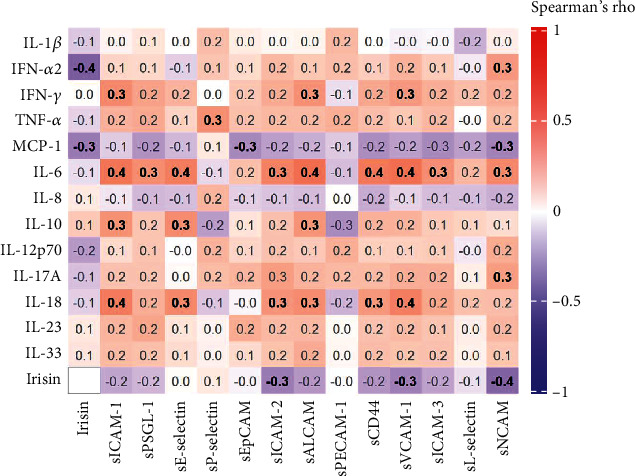
Correlation heat map for irisin and adhesion molecules with cytokine profile in children and adolescents with type 2 diabetes mellitus, metabolic syndrome, and healthy controls. Colored squares of the heat map represent Spearman's rho correlation coefficients; the coefficients' font appears black and bold when correlations are statistically significant and grey when they are not. Squares filled in shades of blue represent negative correlations, while those filled in shades of red represent positive correlations. Statistical significance was established as *p* value ≤ 0.05. IL: interleukin; IFN: interferon; TNF: tumor necrosis factor; MCP: monocyte chemoattractant protein; sICAM: soluble intercellular adhesion molecule; sPSGL: soluble P-selectin glycoprotein ligand; sEpCAM: soluble epithelial cell adhesion molecule; sALCAM: soluble activated leukocyte cell adhesion molecule; sPECAM: soluble platelet endothelial cell adhesion molecule; sVCAM: soluble vascular cell adhesion molecule; sNCAM: soluble neural cell adhesion molecule.

Regarding irisin, the cytokine profile, and cellular adhesion molecules (Figure 3), a significant negative correlation between irisin and MCP-1 (*R* = −0.308, *p* = 0.025), IFN-*α*2 (*R* = −0.406, *p* = 0.003), sNCAM (*R* = −0.382, *p* = 0.005), sICAM-2 (*R* = −0.300, *p* = 0.029), and sVCAM-1 (*R* = −0.292, *p* = 0.034) was found.

When performing a group analysis, irisin was shown to have a positive correlation with the SBP percentile (*R* = 0.545, *p* = 0.024) and a negative association with MCP-1 (*R* = −0.514, *p* = 0.035) in patients with MetS. In this group, a tendency towards a positive correlation of irisin with IL-10 was observed (*R* = 0.445), although not statistically significant (*p* = 0.073). Regarding the T2DM group, a positive correlation was shown between the irisin concentration and TC (*R* = 0.598, *p* = 0.005) as well as LDL-c levels (*R* = 0.550, *p* = 0.012). Complete correlation analyses by groups are presented as Supplementary Tables (available here).

## 4. Discussion

### 4.1. Irisin Concentration in Pediatric Type 2 Diabetes Mellitus and Metabolic Syndrome Patients and Its Relationship with Clinical and Biochemical Parameters

Our results showed lower irisin levels in the group with MetS when compared with healthy controls, as well as an inverse association between the irisin concentration and BMI percentile, WC percentile, and weight. In agreement with our findings, decreased serum irisin levels in adults [32], children, and adolescents [37, 38, 56] have been reported. On the contrary, Jang et al. described increased irisin levels in adolescents with MetS [36]. On the other hand, the irisin concentration has been mostly reported to be decreased in adult patients [30] as well as children and adolescents [39] with T2DM. These findings are in line with our results, as we also observed significant lower serum irisin levels in the T2DM group when compared with healthy controls. Although the exact mechanisms underlying these findings are unclear, PGC-1*α* and *Fndc5* might be involved. In a study evaluating the skeletal muscle, healthy individuals were found to present a fourfold increase of the expression of PGC-1*α* after an acute bout of exercise, while no increase was observed in patients with T2DM [57]. Consistent with this observation, the decreased gene expression of *Fndc5* in adipose tissue of patients with T2DM correlated with a reduction in the plasma irisin concentration [18]. These findings suggest that the lower expression of PGC-1*α* may underlie the lower irisin levels shown in this population. In addition, MetS and T2DM patients share the pathologic features of insulin resistance and low-grade systemic inflammation, which in turn are accompanied with increased concentrations of glucose, free fatty acids, and proinflammatory cytokines. Interestingly, each of these stimuli has been shown to decrease the expression of *Fndc5* in preclinical studies [18, 58, 59].

Results regarding the relationship of irisin and anthropometric parameters are still controversial in pediatric patients. Positive correlations between the serum irisin concentration and BMI, BMI percentile, BF%, and WC have been reported [35, 36, 60, 61]. Our results are in agreement with other studies that have found an inverse association between irisin levels and both BMI and WC in obese children and adolescents [37, 40]. Our observations are also consistent with a study in adults with T2DM that described an inverse association between serum irisin and WC, in which the authors hypothesized that body composition may adversely influence physiological effects of irisin, such as the increase in oxygen consumption and the augmented glucose tolerance [33]. Thus, our results might likely represent a downregulation of irisin by decreased expression of PGC-1*α* and *Fndc5*, attributed to the metabolic dysfunction present in patients with MetS and T2DM.

Regarding clinical and biochemical parameters, studies have also reported contradictory findings. Interestingly, in agreement with a study in adults with MetS [31], our results demonstrated a positive correlation between irisin and the SBP percentile, possibly implying a compensatory response to prevent a further increase in blood pressure. The latter hypothesis is supported by a study in which the irisin treatment decreased blood pressure in a hypertensive rat model via increased nitric oxide production by endothelial cells [23]. On the other hand, we observed no association between irisin levels and the HOMA index. This is a debated topic [31, 33, 35–37, 40], given that controversial results could be related to the already altered insulin sensitivity in patients with MetS and T2DM. Furthermore, our results showed a negative correlation between irisin and triglyceride levels, as has been previously reported in children [37], while positive associations were seen between irisin and TC, LDL-c, and HDL-c levels. The strongest correlation was found with HDL-c, a finding that has been obtained in other studies including patients with T2DM [62] and MetS [63]. This result, along with the known role of HDL-c as an anti-inflammatory and antioxidant lipoprotein [64], suggests that irisin may participate in the endothelial protection in the setting of abnormal glucose homeostasis. Regarding the correlation of irisin with both TC and LDL-c, others have also reported this finding, considering high LDL-c to be one of the strongest predictive factors for high serum irisin concentrations [36]. Thus, this association might also point towards a compensatory mechanism, considering the reported role of irisin in reducing hepatic cholesterol synthesis and lipogenesis [65].

### 4.2. Potential Role of Irisin in the Pathogenesis of Pediatric Type 2 Diabetes Mellitus and Metabolic Syndrome

The significant lower serum irisin concentration observed in both the T2DM and MetS groups might potentially play a pathogenic role in these diseases. Irisin has been shown to increase the expression of UCP1 [16] and to enhance the oxidative metabolism and energy consumption [29]. These effects ultimately represent a stimulus for glucose uptake through increased membrane translocation of GLUT-4 [29]. Therefore, lower irisin levels in the T2DM and MetS groups could reduce the expression of UCP1, decrease oxidative metabolism, and favor hyperglycemia. Moreover, AMP-activated protein kinase (AMPK) has been demonstrated to be another mechanism through which irisin modulates glucose metabolism [66]. AMPK has been reported to inhibit hepatic gluconeogenesis and lipogenesis [66] and to upregulate glucose uptake in the skeletal muscle [67]. Hence, the lower irisin levels observed in both the MetS and T2DM groups could lead to increased gluconeogenesis and lipogenesis, further exacerbating hyperglycemia. The loss of the insulin-sensitizing effect of irisin may represent another deleterious component. In addition, higher irisin concentrations have been shown to favor an anti-inflammatory role by decreasing the membrane expression of TLR4 and downregulating the mitogen-activated protein kinase (MAPK) signaling pathway [25], ultimately decreasing the NF-*κ*B activation [25]. The loss of this anti-inflammatory stimulus due to the lower irisin levels in our T2DM group could play a role in their low-grade systemic inflammation.

### 4.3. Adhesion Molecules and Inflammatory Cytokines in Patients with Type 2 Diabetes Mellitus and Metabolic Syndrome

We observed higher levels of all soluble CAMs in the group with MetS. It is important to note that to the best of our knowledge, no previous studies have reported a comparison including the wide array of adhesion molecules we analysed regarding MetS and T2DM patients in the pediatric population. Previous studies have demonstrated increased levels of sICAM-1 in children [13] and adults with MetS [68, 69], and a positive association between sICAM-1 and insulin resistance was confirmed in children with obesity [12]. Our results showed a positive correlation between sICAM-1, sE-selectin, sICAM-2, sALCAM, sCD44, sVCAM-1, sICAM-3, sL-selectin, and sNCAM with insulin levels and the HOMA index. These findings could indicate that our patients in the MetS group might already present some degree of endothelial dysfunction.

Regarding T2DM, studies involving CAMs in this population have shown contradictory results. Greater levels of sICAM-1 and sVCAM-1 have also been observed in children with T2DM compared with healthy controls [15]. On the other hand, in adult patients with T2DM, serum levels of sL-selectin were lower in comparison with controls [70], while others have described no differences [71–73]. Our results showed no significant difference in the concentration of soluble CAMs in children and adolescents with T2DM compared with healthy controls. Of note, the T2DM group presented lower levels of adhesion molecules when compared with the MetS group. Explanations for these findings are unclear but could suggest the involvement of cellular pathways including microRNAs (miRNAs) [74–77], advanced glycation end-products (AGEs) [78], and reactive oxygen species (ROS) [79]. As described by Jansen et al., the in vitro apoptosis of human coronary artery endothelial cells resulted in the generation of endothelial microparticles (apoptotic bodies) that led to posttranscriptional modifications of ICAM-1 mRNA through translational silencing induced by microRNA- (miR-) 222 [74]. After treatment with TNF-*α*, which increased the ICAM-1 expression, the incubation with endothelial microparticles under hyperglycemic conditions decreased mRNA and protein levels of ICAM-1 and diminished leukocyte adhesion [74]. Likewise, miR-126 was shown to downregulate the translation of VCAM-1 mRNA, resulting in decreased membrane expression of this adhesion molecule [76]. This could explain our findings regarding lower sVCAM-1 levels in the T2DM group when compared with the MetS group. The lower expression of E-selectin and ICAM-1 via miR-31 and miR-17-3p, respectively, which are induced by TNF-*α*, has been related to a decrease in adhesion of neutrophils to endothelial cells [77]. Therefore, given our results, we hypothesize that the development of overt T2DM in the pediatric population might possibly cause an increase of their low-grade systemic inflammation, with subsequent endothelial cell apoptosis that generates endothelial microparticles. These microparticles, through augmented miRNA expression, might trigger counterregulatory or compensatory mechanisms that ultimately result in the downregulation of the expression of soluble CAMs, when compared with patients with MetS that have not yet developed T2DM. In addition, hyperglycemia has been proven to accelerate the production of AGEs that activate inflammatory pathways by engaging AGE receptors (RAGEs) in the vascular endothelium [78] and macrophages [80]. Furthermore, hyperglycemia has been shown to activate AGE-independent deleterious pathways that result in the endothelial oxidative stress and apoptosis [79]. Hence, the effect of important endothelial apoptosis may not yet be observed in patients with MetS without T2DM, possibly due to relatively lower levels of oxidative stress, when compared with overt T2DM subjects. Thus, it may be hypothesized that higher levels of ROS observed in T2DM, generated by multiple stressors (RAGE and TNF-R signaling, mitochondrial dysfunction, lipotoxicity, etc.), induce apoptosis, while lower ROS concentrations present in MetS engage instead in pathways triggered by the activation of NF-*κ*B, which increases antiapoptotic molecules [81].

Furthermore, we observed lower levels of sPECAM-1 in T2DM patients compared with the MetS group. PECAM-1 has been described to be degraded by matrix metalloproteinase-2 (MMP-2), an enzyme found to be upregulated by hyperglycemia [82]. This represents another regulatory pathway that may limit the expression of adhesion molecules in our patients with T2DM. AGEs could also downregulate PECAM-1, as endothelial cells that have been treated with AGEs and TNF-*α* showed a more prominent decrease in the PECAM-1 mRNA expression compared with endothelial cells only treated with TNF-*α*, but no change in expression was seen when cells were only treated with AGEs [83]. Therefore, these results might imply that AGEs permissively enhance the PECAM-1 downregulation only in the presence of a proinflammatory milieu.

In the context of T2DM, proinflammatory cytokines have been shown to contribute to endothelial injury in association with increased glucose levels. Our results showed significant higher levels of the proinflammatory cytokines MCP-1 and IL-18 and a tendency toward higher IL-8 levels in the T2DM group. In relation to these cytokines, experiments performed in human retinal endothelial cells demonstrated that diabetes-related endothelial injury was mediated by glucose-induced proinflammatory cytokine elevation, leading to the apoptosis of endothelial cells [84]. IL-1*β* and TNF-*α* have been shown to stimulate the secretion of other proinflammatory cytokines, such as IL-8 and MCP-1 by endothelial cells [84], while the caspase-1 activation has been demonstrated to produce IL-1*β* and IL-18 [85]. These mechanisms of cytokine secretion might thus be present in our patients with T2DM. Higher concentrations of MCP-1 and IL-18 in the context of T2DM have been shown in adult patients [86, 87]. MCP-1 has been described to be released by adipocytes in response to hyperglycemia [88] and has been considered an important driver of monocyte influx into sites of inflammation [8]. Overall, both inflammation and hyperglycemia promote a compensatory response consisting of lower expression of endothelial CAMs to counteract and limit inflammation, at least in the early stages of metabolic disease, as what can be seen in our young patients. Moreover, MCP-1 and IL-18 are involved in the leukocyte influx into adipose tissue, representing a perpetuating cycle of inflammation driven by Th1 lymphocytes; this pathway constitutes a primary initial step in the early stages of atherogenesis [8, 88, 89]. Furthermore, the reasons for the lack of significant higher values of TNF-*α*, IL-1*β*, and IL-6 in the T2DM and MetS groups are unclear but might involve technical aspects in their measurement [90–92].

### 4.4. Irisin and Its Association with Endothelial Dysfunction and Inflammatory Markers

The relationship between irisin, adhesion molecules, and endothelial dysfunction may be a relevant component in the pathophysiology of vascular damage in MetS and T2DM. Our results showed lower irisin levels in the MetS and T2DM groups when compared with healthy controls. In vitro and animal studies have shown that irisin decreases the endothelial dysfunction by upregulating endothelial nitric oxide synthase (eNOS) and phosphoinositide-3-kinase (PI3K)/Akt phosphorylation and activation [93]. It also reduces endothelial dysfunction by downregulating the protein kinase C-*β*/nicotinamide adenine dinucleotide phosphate (PKC-*β*/NADPH) and NF-*κ*B/inducible nitric oxide synthase (iNOS) pathways [22]. The results reported in these studies might suggest that the protective role of irisin in decreasing endothelial dysfunction might be altered in our pediatric patients with MetS and T2DM. Of note, there is no report to date evaluating a wide array of molecules such as the panel used in this study in children and adolescents diagnosed with MetS or T2DM. In this regard, our results showed a negative correlation between irisin and sVCAM-1, sICAM-2, and sNCAM. Yin and colleagues reported an inverse association between irisin and E-selectin and ICAM-1, but no correlation with VCAM-1 in children with obesity [40]. It is important to highlight that this study did not include patients diagnosed with T2DM or MetS. We then hypothesize that low irisin levels may potentially contribute to the higher levels of soluble CAMs observed in the MetS group. On the other hand, in the context of T2DM, other mechanisms may be responsible for the downregulation of these adhesion molecules as a compensatory mechanism. Considering that irisin has been described to protect against endothelial oxidative stress [22, 94], low levels of irisin in the T2DM group may allow the loss of this antioxidant mechanism. Additionally, endothelial cell apoptosis in the context of T2DM induced by ROS [79], AGEs [78], and glucotoxicity [78, 80] could then trigger the release of miRNAs that reduce the expression of CAMs [74–77]. This effect may not be observed in patients with MetS, theoretically due to the lower glucotoxicity, AGEs, and ROS (as opposed to what is seen in T2DM patients), with a subsequent tendency for endothelial cell activation, instead of apoptosis. Consequently, this endothelial cell activation upregulates the expression of adhesion molecules [95] in patients with MetS.

We observed an inverse correlation between the serum irisin concentration and both MCP-1 and IFN-*α*2 considering the total population of children and adolescents. When analysing the MetS group by itself, a negative association with MCP-1 was also present, while a tendency towards a significant positive correlation of irisin with the anti-inflammatory cytokine IL-10 was observed. Irisin decreases the expression and secretion of the proinflammatory cytokines TNF-*α*, IL-1*β*, IL-6, and MCP-1 in macrophages by modulating TLR4/NF-*κ*B signaling [25]. Altogether, the negative association between irisin and MCP-1, as well as the fact that our patients presented hypoirisinemia, could potentially suggest a loss of this adipomyokine's anti-inflammatory role. In addition, irisin has been found to promote the M2 macrophage polarization [27, 28], which in turn induces the IL-10 production [96]. The observed tendency for IL-10 to be positively associated with irisin in MetS patients might suggest that the hypoirisinemia seen in this group may represent a lack of stimulus for the macrophage M2 polarization and IL-10 production. On the other hand, it has been proposed that type I interferons, including IFN-*α*2, might play an important role in the infiltration of CD8+ T cells into the liver [97] and adipose tissue [98, 99]. The inverse relationship between irisin levels and IFN-*α*2 shown in our results might imply that the lower irisin levels observed in patients with MetS and T2DM are not capable of counteracting the CD8+ T cell infiltration into the liver and adipose tissue.

Overall, our results regarding the inverse relationship between irisin and sNCAM, sICAM-2, sVCAM, MCP-1, and IFN-*α*2, as well as the pathogenic role that these molecules have in the progression of MetS and T2DM, lead us to hypothesize that the low irisin levels observed in our patients might blunt the downregulating effects of irisin towards these inflammatory and cell adhesion molecules.

### 4.5. An Integrated Overview of the Hypothesized Interplay between Irisin, Inflammation, and Endothelial Dysfunction in Pediatric Patients with Type 2 Diabetes Mellitus and Metabolic Syndrome

**Figure 4:**
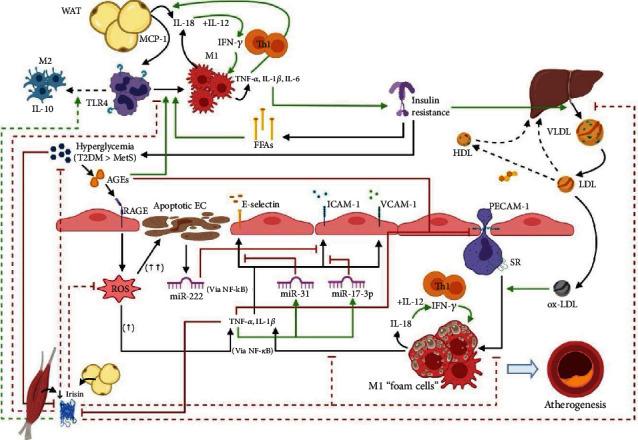
Proposed interplay between low irisin levels, endothelial cell adhesion molecules, and inflammatory cytokines in the context of type 2 diabetes mellitus and metabolic syndrome. See text for more detailed information. AGEs: advanced glycation end-products; EC: endothelial cell; FFAs: free fatty acids; HDL: high-density lipoprotein; ICAM-1: intercellular adhesion molecule-1; IFN-*γ*: interferon gamma; IL-1*β*: interleukin-1 beta; IL-6: interleukin-6; IL-10: interleukin-10; IL-12: interleukin-12; LDL: low-density lipoprotein; MCP-1: monocyte chemoattractant protein-1; MetS: metabolic syndrome; miR: microRNA; NF-*κ*B: nuclear factor-kappa B; ox-LDL: oxidized low-density lipoprotein; PECAM-1: platelet endothelial cell adhesion molecule; RAGE: receptor for advanced glycation end-products; ROS: reactive oxygen species; SR: scavenger receptor; T2DM: type 2 diabetes mellitus; Th1: T-helper 1 cell; TLR4: toll-like receptor 4; TNF-*α*: tumor necrosis factor-alpha; VCAM-1: vascular cell adhesion protein-1; VLDL: very low-density lipoprotein; WAT: white adipose tissue. This is an original figure created with the BioRender® platform (BioRender, Toronto, ON, Canada), accessed in May 2020 through the following URL: https://biorender.com/, with a premium member's account [A01191497@itesm.mx].

Our proposed view of the interplay between irisin, proinflammatory cytokines, and endothelial dysfunction in the context of pediatric MetS and T2DM is integrated in Figure 4. In summary, hypoirisinemia, possibly triggered by hyperglycemia-induced stress [18, 58], could promote an exacerbation of the low-grade systemic inflammatory state. This state might be attributed to the lack of inhibition of proinflammatory cytokine synthesis by the adipose tissue macrophages and reduced M2 polarization [27, 28]. This microenvironment promotes insulin resistance, resulting in increased circulating free fatty acids and hyperglycemia [100]. Free fatty acids exacerbate the inflammatory response by engaging TLR4 in macrophages [8], while hyperglycemia accelerates the production of AGEs, which also elicit the M1 macrophage polarization [80]. Inflammatory cytokines drive the upregulation of endothelial CAMs such as VCAM-1, ICAM-1, and E-selectin, through the activation of NF-*κ*B [9], thus favoring an influx of inflammatory cells into the vascular intima and the progression of vascular disease. Notably, TNF-*α* may also induce the downregulation of CAMs by means of miRNAs as a negative feedback mechanism to limit inflammation [77] in the context of T2DM. Considering that hyperglycemia is higher in T2DM, AGEs could engage endothelial RAGEs to a greater degree in T2DM than in MetS. Hence, an increase of the endothelial oxidative stress could trigger significant endothelial cell apoptosis [81] that leads to the release of microparticles containing miRNAs. These miRNAs downregulate endothelial CAMs [74], further limiting inflammatory cell influx as a compensatory mechanism against the progression of vascular damage that is observed in early stages of this disease. On the other hand, the endothelial ROS generation may be relatively lower in MetS when compared with T2DM, which could be insufficient to trigger a significant amount of endothelial cell apoptosis. Consequently, a lack of inhibition of endothelial CAMs might ensue. Moreover, insulin resistance produces increased lipogenesis in the liver and an unfavorable lipid profile that stimulates atherosclerotic plaque formation [6], further contributing to vascular damage. Additionally, low irisin levels promote the lipid accumulation and apoptosis of foam cells [101], the exacerbation of proinflammatory cytokine secretion [24], and the endothelial ROS production [22, 94], all of which contribute to plaque growth and destabilization. Finally, IL-18, in the presence of IL-12, maintains a proinflammatory microenvironment driven by Th1 cells in both the adipose tissue and the vasculature [89].

There are some limitations present in our study. All of the patients are of Hispanic origin; therefore, our conclusions may not apply to other populations and must be interpreted with caution. Importantly, this is a cross-sectional study; hence, our results should be viewed as associations and cannot be used to draw definite conclusions regarding cause-and-effect relationships. On the other hand, one of our strengths is that to the best of our knowledge, this is the first study that analyses pediatric patients diagnosed with MetS and T2DM, as well as healthy controls in order to assess the association of irisin with a wide array of soluble adhesion molecules and inflammatory cytokines. The analysis of this information is also integrated in an original figure (Figure 4), where the interplay of the results of in vitro and animal model studies are represented to try to further clarify the molecular mechanisms that might be implicated in our results. Finally, all of the information presented in this study is completely objective, as no questionnaires or other subjective methods were used.

## 5. Conclusions

Obesity and its related metabolic diseases, including T2DM and MetS, in the pediatric population are a worldwide public health concern with an increasing prevalence and incidence within this age group. The role of irisin, a novel adipomyokine, is controversial in children and adolescents and has been scarcely studied in MetS and T2DM, as well as its association with CAMs and inflammation. To the best of our knowledge, this is the first study evaluating the association between irisin and a wide array of inflammatory cytokines and adhesion molecules in the context of pediatric patients with T2DM and MetS. Our results showed significant lower serum irisin levels in both MetS and T2DM patients compared with healthy controls. Decreased concentrations of sICAM-1, sPSGL-1, sEpCAM, sICAM-2, sALCAM, sCD44, sVCAM-1, sICAM-3, sL-selectin, sNCAM, sP-selectin, and sPECAM-1 were found in the T2DM group when compared with MetS patients. In addition, higher concentrations of MCP-1 and IL-18 were observed in the T2DM group compared with healthy controls. Irisin was negatively correlated with sNCAM, sVCAM, sICAM-2, IFN-*α*2, and MCP-1, while sICAM-1, sE-selectin, sICAM-2, sALCAM, sCD44, sVCAM-1, sICAM-3, sL-selectin, and sNCAM correlated positively with BMI and WC percentiles, the HOMA index, and insulin levels. Metabolic stressors such as the hyperglycemic state seen in MetS and T2DM patients might be able to induce hypoirisinemia through a decrease in the *Fndc5* expression. We hypothesize that hypoirisinemia may increase ROS and favor a proinflammatory state mediated by an increase in the TLR4 expression, which results in the NF-*κ*B activation. In addition, in an overt diabetic state, other mechanisms such as ROS, AGEs, and glucotoxicity trigger endothelial cell apoptosis, with consequent production of miRNA-containing microparticles. These miRNAs eventually lead to a downregulation of endothelial CAMs as a compensatory mechanism to prevent further progression of vascular damage. Considering that the levels of inflammation and oxidative stress in children and adolescents with MetS might be lower than those present in T2DM, the hypoirisinemia seen in MetS patients might promote an upregulation of CAMs. Further research on the association of irisin and vascular complications in pediatric patients with MetS and T2DM are needed, including in vitro and experimental models, to better clarify and understand the mechanisms underlying the pathophysiological role of irisin in these metabolic diseases.

## Figures and Tables

**Table 1:** Demographic, anthropometric, and clinical characteristics of children and adolescents with type 2 diabetes mellitus, metabolic syndrome, and healthy controls.

	Healthy controls (*n* = 16)	Metabolic syndrome (*n* = 19)	Type 2 diabetes mellitus (*n* = 21)	*p* value
Age (years)	11.4 (±2.8)^c^	12.4 (±2.1)	13.8 (±1.5)^a^	0.0040
Weight (kg)	37.6 (29.2-46.2)^b,c^	71.1 (52.8-82.1)^a^	67.1 (61.4-87.4)^a^	<0.0010
Height (cm)	145.0 (133.0-159.3)^c^	154.0 (150.0-165.0)	159.0 (155.0-165.0)^a^	0.0190
BMI (kg/m^2^)	17.8 (16.4-18.5)^b,c^	26.0 (24.0-31.6)^a^	27.7 (25.8-31.0)^a^	<0.0010
BMIp	60.0 (41.8-68.8)^b,c^	98.0 (93.0-99.0)^a^	96.0 (93.0-99.0)^a^	<0.0010
WC (cm)	64.1 (±6.0)^b,c^	89.0 (±12.4)^a^	95.7 (±16.4)^a^	<0.0010
WCp	25.0 (15.0-25.0)^b,c^	85.0 (75.0-90.0)^a^	85.0 (75.0-95.0)^a^	<0.0010
Hip (cm)	66.0 (±6.5)^b,c^	96.8 (±12.7)^a^	101.9 (±16.5)^a^	<0.0010
WC-to-hip ratio	1.0 (±0.0)	0.9 (±0.1)	0.9 (±0.1)	0.1180
WC-to-height ratio	0.4 (±0.0)^b,c^	0.6 (±0.1)^a^	0.6 (±0.1)^a^	<0.0010
SBP (mmHg)	99.0 (96.0-104.0)^b,c^	114.0 (101.5-117.0)^a^	113.0 (108.0-120.0)^a^	<0.0010
SBPp	38.0 (28.8-40.5)	79.0 (29.5-88.5)	62.0 (36.0-90.0)	0.0530
DBP (mmHg)	64.0 (63.5-66.5)	66.0 (62.5-71.5)	68.0 (63.0-80.0)	0.3390
DBPp	61.6 (±8.9)	60.6 (±26.8)	65.2 (±25.7)	0.7980

Data are presented as mean (±standard deviation) for parametric data and as median (interquartile range) for nonparametric data. ^a^Statistical difference when compared vs. healthy control group; ^b^statistical difference when compared vs. metabolic syndrome group; ^c^statistical difference when compared vs. type 2 diabetes mellitus group. BMI: body mass index; BMIp: BMI percentile; WC: waist circumference; WCp: waist circumference percentile; SBP: systolic blood pressure; SBPp: systolic blood pressure percentile; DBP: diastolic blood pressure; DBPp: diastolic blood pressure percentile. Statistical difference was established as *p* value ≤ 0.05.

**Table 2:** Biochemical parameters in children and adolescents with type 2 diabetes mellitus, metabolic syndrome, and healthy controls.

	Healthy controls (*n* = 16)	Metabolic syndrome (*n* = 19)	Type 2 diabetes mellitus (*n* = 21)	*p* value
Irisin (ng/mL)	30.3 (24.6-57.1)^b,c^	6.6 (2.8-18.0)^a^	6.8 (2.2-23.2)^a^	0.0040
Glucose (mg/dL)	87.0 (79.0-89.0)^b,c^	94.0 (89.5-103.5)^a^	115.0 (92.0-169.0)^a^	<0.0010
Insulin (mIU/L)	6.6 (5.4-7.9)^b,c^	20.1 (15.3-25.8)^a^	22.9 (10.6-29.8)^a^	<0.0010
HOMA index	1.3 (±0.4)^b,c^	5.4 (±2.2)^a,c^	9.1 (±7.0)^a,b^	<0.0010
TC (mg/dL)	151.0 (±21.3)	159.8 (±33.2)	149.1 (±28.6)	0.4600
TG (mg/dL)	90.0 (65.8-98.5)^b,c^	144.0 (128.0-213.5)^a^	154.0 (112.0-187.0)^a^	<0.0010
HDL-c (mg/dL)	53.8 (±13.0)^b,c^	38.6 (±7.8)^a^	37.2 (±7.9)^a^	<0.0010
LDL-c (mg/dL)	98.6 (±23.8)^c^	87.6 (±27.0)	75.9 (±20.6)^a^	0.0210

Data are presented as mean (±standard deviation) for parametric data and as median (interquartile range) for nonparametric data. ^a^Statistical difference when compared vs. healthy controls group; ^b^statistical difference when compared vs. metabolic syndrome group; ^c^statistical difference when compared vs. type 2 diabetes mellitus group. HOMA: Homeostatic Model Assessment; TC: total cholesterol; TG: triglycerides; HDL-c: high-density lipoprotein cholesterol; LDL-c: low-density lipoprotein cholesterol. Statistical difference was established as *p* value ≤ 0.05.

**Table 3:** Adhesion molecule profile in children and adolescents with type 2 diabetes mellitus, metabolic syndrome, and healthy controls.

Concentration (pg/mL)	Healthy controls (*n* = 16)	Metabolic syndrome (*n* = 19)	Type 2 diabetes mellitus (*n* = 21)	*p* value
sICAM-1	122.2 (49.7-230.0)^b^	653.7 (276.8-808.1)^a,c^	146.5 (93.5-337.4)^b^	<0.0001
sPSGL-1	3.3 (1.8-4.7)^b^	5.8 (4.0-8.9)^a,c^	3.5 (2.4-5.6)^b^	0.0021
sE-selectin	62.3 (28.1-115.4)^b^	177.0 (64.7-316.9)^a^	64.2 (53.7-114.6)	0.0238
sP-selectin	146.4 (48.1-575.2)	319.9 (131.4-648.0)^c^	107.9 (107.9-253.8)^b^	0.0180
sEpCAM	0.1 (0.1-0.2)^b^	0.4 (0.1-0.8)^a,c^	0.1 (0.1-0.3)^b^	0.0152
sICAM-2	42.5 (17.1-81.1)^b^	254.8 (137.8-300.1)^a,c^	59.3 (34.6-113.5)^b^	<0.0001
sALCAM	59.8 (26.6-102.8)^b^	256.0 (150.8-350.2)^a,c^	80.4 (45.0-134.1)^b^	<0.0001
sPECAM-1	61.7 (21.7-92.9)	96.0 (52.0-154.3)^c^	41.7 (15.7-64.7)^b^	0.0021
sCD44	110.1 (62.3-192.0)^b^	389.6 (290.2-421.7)^a,c^	145.1 (84.3-207.5)^b^	<0.0001
sVCAM-1	740.4 (426.9-1121.0)^a^	2887.0 (2305.0-4893.0)^a,c^	1201.0 (727.9-1682.0)^b^	<0.0001
sICAM-3	51.2 (26.7-70.5)^b^	133.2 (102.8-197.6)^a,c^	48.4 (30.9-92.2)^b^	<0.0001
sL-selectin	2634.0 (1203.0-3759.0)^b^	5818.0 (3590.0-9547.0)^a,c^	2472.0 (1664.0-518.0)^b^	0.0002
sNCAM	93.3 (69.1-171.1)^b^	397.2 (320.4-632.3)^a,c^	127.7 (64.3-201.7)^b^	<0.0001

Data are presented as median (interquartile range) for nonparametric data. ^a^Statistical difference when compared vs. healthy controls group; ^b^statistical difference when compared vs. metabolic syndrome group; ^c^statistical difference when compared vs. type 2 diabetes mellitus group. ICAM: intercellular adhesion molecule; PSGL: P-selectin glycoprotein ligand; EpCAM: epithelial cell adhesion molecule; ALCAM: activated leukocyte cell adhesion molecule; PECAM: platelet endothelial cell adhesion molecule; VCAM: vascular cell adhesion molecule; NCAM: neural cell adhesion molecule. Statistical difference was established as *p* value ≤ 0.05.

**Table 4:** Cytokine profile in children and adolescents with type 2 diabetes mellitus, metabolic syndrome, and healthy controls.

Concentration (pg/mL)	Healthy controls (*n* = 16)	Metabolic syndrome (*n* = 19)	Type 2 diabetes mellitus (*n* = 21)	*p* value
IL-1*β*	0.8 (0.1-1.9)	0.6 (0.3-1.9)	0.9 (0.5-2.7)	0.5264
IFN-*α*2	0.4 (0.1-3.8)	3.8 (0.2-12.9)	1.5 (0.2-5.4)	0.0936
IFN-ɣ	2.2 (0.3-4.4)	3.5 (1.4-5.0)	4.0 (1.7-5.3)	0.2004
TNF-*α*	1.4 (0.5-2.9)	1.7 (0.9-2.3)	1.4 (0.7-2.0)	0.8198
MCP-1	197.2 (±38.6)^c^	215.7 (±65.7)	258.2 (±70.4)^a^	0.0108
IL-6	2.5 (1.3-4.5)	3.7 (1.7-4.6)	3.7 (2.2-5.0)	0.2763
IL-8	4.6 (2.4-7.0)	6.0 (2.9-8.9)	7.8 (5.4-12.5)	0.0516
IL-10	1.6 (±1.1)	1.7 (±0.9)	2.2 (±1.0)	0.1528
IL-12p70	0.4 (0.1-0.8)	0.5 (0.2-1.4)	0.6 (0.2-1.1)	0.6901
IL-17A	6.7 (1.5-11.7)	13.8 (5.1-21.5)	6.6 (4.7-16.5)	0.1451
IL-18	139.6 (102.6-177.2)^c^	203.0 (119.8-246.1)	216.2 (169.2-257.4)^a^	0.0027
IL-23	4.4 (2.3-6.7)	4.4 (3.0-8.2)	6.7 (2.4-8.3)	0.6348
IL-33	8.3 (2.3-12.0)	7.3 (4.1-21.4)	10.1 (5.2-15.2)	0.4732

Data are presented as median (interquartile range) for nonparametric data and as mean (±standard deviation) for parametric data. ^a^Statistical difference when compared vs. healthy controls group; ^b^statistical difference when compared vs. metabolic syndrome group; ^c^statistical difference when compared vs. type 2 diabetes mellitus. IL: interleukin; IFN: interferon; TNF: tumor necrosis factor; MCP: monocyte chemoattractant protein. Statistical difference was established as *p* value ≤ 0.05.

## Data Availability

Tables with the complete correlation analysis for T2DM, MetS, and healthy control groups are included within the supplementary information files. Patients and their legal guardians signed an informed consent allowing our research team to use their clinical and biochemical data and so cannot be publicly disclosed. Requests for access to these data should be made to the corresponding author Leticia Elizondo-Montemayor (lelizond@tec.mx).

## Supplementary Materials

Supplementary Materials A complete correlation analysis for type 2 diabetes mellitus, metabolic syndrome, and healthy controls groups is attached as Supplementary Tables. Spearman's rho correlation coefficients for all variables included in this study are presented. There is an individual table with correlations for each of the study groups. ^∗^*p* < 0.05; ^∗∗^*p* < 0.01; ^∗∗∗^*p* < 0.001.
